# Birds of Berlin: Changes in communities and guilds in the urban park “Tiergarten” since 1850

**DOI:** 10.1002/ece3.11461

**Published:** 2024-05-26

**Authors:** Esther Sophie Felgentreff, Nadja Pernat, Sascha Buchholz

**Affiliations:** ^1^ Department of Ecology and Evolution Friedrich‐Schiller‐Universität Jena Jena Germany; ^2^ Institute of Landscape Ecology University of Münster Münster Germany

**Keywords:** community ecology, functional diversity, long‐term study, ornithology, urban ecology, biodiversity

## Abstract

Urbanization has far‐reaching consequences on birds, and knowledge of the impacts on taxonomic and functional diversity is necessary to make cities as compatible as possible for species. Avian diversity in parks in urban centers has been investigated multiple times, but rarely so in long‐term studies due to lacking data. The Tiergarten in Berlin is a large‐scale park in the city center of great value for people and many species including birds. We compiled bird species lists since 1850 and from monitoring in 2022 in one dataset to investigate how bird communities and guilds have changed over time and how these alterations were influenced by the eventful history of the park's vegetation conditions. Long‐term changes in species assemblages were analyzed with an ordination analysis, and changes in guild presence and functional richness were discussed with regard to landscape transitions. A gradual development of species assemblages yet only small changes in guild composition since 1850 was detected, whereas the 1950 community stands out with a drop in species richness and replacement of forest species with an open land community, which reflects the deforestation of the park during World War II. Consideration of habitat, lifestyle, trophic, and migration guilds revealed no sign of functional homogenization over the last 172 years (1850–2022). Despite the high frequentation of the park by humans it still allows for a high bird diversity due to the Tiergarten's sheer size and heterogeneity of vegetation and habitats. We recommend that the park is maintained and managed accordingly to preserve this condition and advise other urban parks to strive for these beneficial features.

## 1 INTRODUCTION

Human's impact on the world's ecosystems is far‐reaching (Vitousek et al., 1997), and urbanization is one of the most drastic changes made on terrestrial lands (Grimm et al., 2008). Currently, over 50% of the world's population resides in cities, a number which is projected to increase steadily (UN DESA, 2019), and urbanized areas are the fastest‐growing habitats in the world (Grimm et al., 2008). Urban areas differ from natural areas in many ways, creating special conditions for species. These include abiotic factors, such as different climatic conditions (Oke, 1973), modified hydrological processes (Paul & Meyer, 2001), and disturbances like noise (EEA, 2020), artificial light (Hölker et al., 2010), and air pollution (UBA, 2019). Species thus find a highly fragmented and heterogeneous landscape with numerous disturbance factors and, compared to natural areas, fewer habitats due to the limited availability of green spaces and high proportion of built‐up areas (Faeth et al., 2012). In addition, novel biotic interactions resulting from the introduction of non‐native species, a decline in natives, and thus an overall change in species composition contribute to the novelty of urban ecosystems (Faeth et al., 2012).

Consequently, urbanization also affects biodiversity but findings are still ambiguous. Some studies report an overall negative impact (Aronson et al., 2014; Grimm et al., 2008; Ibáñez‐Álamo et al., 2017), whereas others give evidence that urban areas can harbor higher diversity than their surroundings (i.e., Gagné et al., 2016; Imai & Nakashizuka, 2010; Ives et al., 2016; Thompson et al., 2022). In any case, scholars agree that especially urban green spaces play an important role in supporting urban biodiversity (i.e., Fontana et al., 2011; Oliveira Hagen et al., 2017; Sandström et al., 2006).

Distinct bird communities exist in cities, some with high diversity (Aronson et al., 2014), but birds are strongly affected by urbanization (Marzluff, 2017). Birds are advantageous in studies on the effect of disturbances and environmental changes because they are comparably easy to monitor, and play important roles in the functioning of ecosystems (Sekercioglu, 2006; Sol et al., 2020). Urban parks can serve as valuable habitats for birds, especially when they cover a large area and feature structural diversity (Morelli et al., 2018). However, how well birds can cope with urban conditions is dependent on their functional traits. These refer to the resources birds require, such as their diet or habitat, and in turn to their effects on ecosystems. Depending on their traits and their behavior towards urbanization, birds can be classified as urban exploiters, adapters, and avoiders (McKinney, 2002). Species that benefit from urbanization are classified as urban exploiters. Examples of exploiters are omnivore or granivore birds that can make use of human structures and food for shelter, breeding, and feeding, as well as species that forage on the ground and nest in artificial structures, e.g., concrete walls that resemble rocky habitats. Adapters include birds of many different feeding guilds that profit from different urban characteristics, such as granivores benefiting from bird feeders, insectivores feeding on fertilized lawns, and aerial sweepers making use of insects attracted by lights. Species sensitive to disturbances that traditionally inhabit forest interiors and nest on the ground belong to the urban avoiders group (McKinney, 2002).

Although there is a growing number of studies on avian diversity in general, not much research has been conducted on the diversity of functional traits (La Sorte et al., 2018; Marzluff, 2017; Matuoka et al., 2020). Functional traits play a crucial role, as they affect birds' primary functions in ecosystems, and therefore, contribute to their services and resilience. For example, ecosystem services like pollination, seed dispersal, and insect/pest control are determined by feeding habits (Matuoka et al., 2020; Sekercioglu, 2006). Studies investigating functional diversity found a loss of specialists and replacement with generalists in urban areas (Callaghan et al., 2023; Marzluff, 2017; Yang et al., 2023), which leads to the reduction of trait guilds occupied in an ecosystem that can threaten its stability by losses of links and functions (Matuoka et al., 2020; Sekercioglu, 2006). Besides taxonomic approaches, it is therefore worthwhile to include trait analyses in a study on bird diversity to gain a better understanding of functional patterns. Long‐term studies following changes in functional bird diversity are especially required to understand the implications of temporal shifts therein (Sekercioglu, 2006). A large number of studies on birds' responses to urbanization use spatial comparisons, for example, by employing urbanization gradients: In their review of choice and frequency of urbanization metrics in studies, Moll et al. (2019) point out that dynamic metrics are widely understudied, despite their high potential meaningfulness. Investigating dynamic metrics means observing changes in environmental or disturbance factors over time and corresponding species responses. As urbanization is characterized by drastic and often quick changes, it is of central importance to understand the effects of these dynamic changes on species when aiming to understand species' response to urbanization patterns and draw conclusions for urban planning (Pickett et al., 2011; Ramalho & Hobbs, 2012). Some studies have applied a space‐for‐time substitution where insights from spatial changes over an urbanization gradient are used for assumptions on temporal changes, but it is questionable whether space can be a sufficient proxy for time (La Sorte et al., 2018).

In this study, we investigated the temporal dynamics of bird communities and their different traits in the Großer Tiergarten (hereafter ‘Tiergarten’) of Berlin. The Tiergarten is the second largest inner‐city park in Germany with detailed bird data and species lists from the 19th century to today. The data allow for a long‐term study to gain insights into the implications of dynamic urbanization on bird diversity. Moreover, due to the eventful history of Berlin and corresponding, Tiergarten, the past land‐use changes of the park allow the consideration of different parameters that influence bird communities over time, namely vegetation, the degree of urbanization, and the frequentation and use of the park. Specifically, we investigated how bird communities and the presence of feeding, habitat, lifestyle, and migratory guilds in Tiergarten have changed since 1850 by merging available historic and recent species lists. Based on this dataset compilation, we assigned corresponding guilds and investigated (i) the current bird community and the changes (ii) in species communities and (iii) in guild communities over time. The temporal component of this study contributes to a better understanding of bird community patterns and influencing factors in a highly dynamic, intensely urbanized green space.

## 2 MATERIALS AND METHODS

### 2.1 Study area

Berlin (52.31° N, 13.24° E) is the capital and largest city of Germany, with an area of just over 891 km^2^ (Amt für Statistik Berlin‐Brandenburg, 2019) and a population of almost 3.8 mio. people (Amt für Statistik Berlin Brandenburg, 2023). The area is characterized by a warm temperate continental climate (on the transition zone to a maritime climate), CfB according to the Köppen climate classification, with mean annual temperatures of around 9.5°C (DWD, 2013a) and mean annual precipitation of 591 mL (DWD, 2013b). Berlin holds many green areas, from highly frequented parks to forests which together make up over 168 km^2^, almost 20% of the city area (Amt für Statistik Berlin‐Brandenburg, 2019).

The Tiergarten is a vast, over 200 ha large park right in the center of Berlin, carrying a unique history: First created in the 16th century as hunting grounds, it was then turned into a forest park for recreational purposes for the town population in the 18th century – while being subject to ever‐changing landscape planning designs in the course of time. During World War II the park was heavily bombed, and much of the remaining groves were used for heating in the post‐war era (Wendland, 1993), leading to an (almost) open area for the first time. It was reforested in the 1950s (Sprötge, 1991). During the division of Berlin, it lay in the periphery of Westberlin rather than in the city center, but after the fall of the wall, it was situated right in the middle of Berlin again. Today, the Tiergarten is mostly covered with forest, but also features open grassy areas. It is highly frequented by Berliners and tourists for recreational purposes. In some parts, the park is crossed by creeks, which sporadically form water bodies, some of which contain islands. The Tiergarten is furthermore crossed by several large and busy streets that divide it into smaller parts, which differ in their character, vegetation, and frequentation. The Tiergarten is an advantageous study location due to its size, its high‐structural diversity, and the availability of detailed breeding bird and habitat characteristic data from over 170 years.

### 2.2 Bird data

The historical species lists originate from Sprötge (1991) who compiled the results from historical bird mappings between 1850 and 1960 in a comprehensive species list. Other existing bird species lists from the Tiergarten were omitted from the analyses as they include only breeding species. Namely, that are lists resulting from monitorings in 1978 by Anders (as cited by Sprötge et al., 1991), in 1988 by Sprötge (1991) and 2010 by Scharon (2010). All of the species lists included in the analyses contain all bird species encountered in Tiergarten, including guests.

**FIGURE 1:**
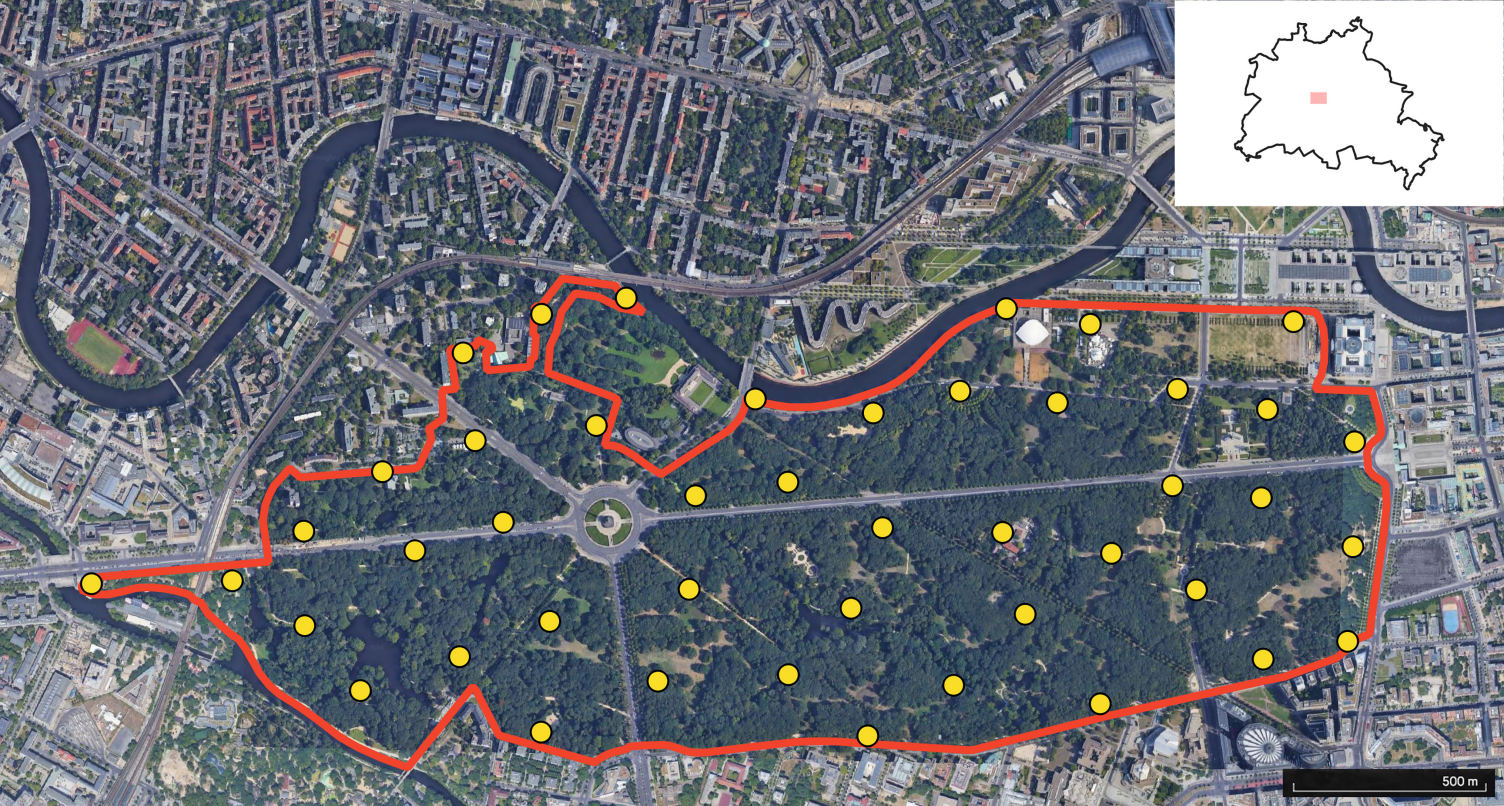
The Großer Tiergarten park in Berlin. The red line marks the site boundary. The yellow dots mark the point count locations. The small additional map depicts the park's location in Berlin. Modified Google Earth Satellite Image (Google Earth, 2020).

To obtain bird community data from 2022, birds were monitored following Fischer et al.'s (2005) commonly used method of point stop counts. This method requires five control walks per monitoring area in different time frames, in which the mapper walks through the area, always following the same route. On predefined points, the mapper stops for exactly 5 min and notes all birds that are visually and/or acoustically detectable, writing down the maximum count of individuals of a species detected at the same time. When arriving at a point, standing still for 20 s before starting the 5‐min‐counts allows birds to settle after the disturbance of approaching – an optional modification suggested by Bibby et al. (1995). The points should cover the extent of the area including all its (different) habitats and habitat borders and were set randomly using GIS. We adjusted the minimum distance between single points to 200 m as recommended by Bibby et al. (1995) rather than the 300 m suggested by Fischer et al. (2005). Monitoring happened between March and June 2022 (week of March 21–25, April 18–22, May 2–6, May 23–27, and June 6–10, Fischer et al., 2005). We split the park into three parts so that three monitoring walks were needed to cover the whole park, resulting in 15 monitoring walks in total. In each week, the three routes were walked on Tuesdays, Thursdays, and Fridays in the same order. Starting at the first point at sunrise, each route took ca. 2.5 h, so the earliest start was at 4:45 am and the latest ending at 8:30 am. Counting was not done on days with heavy wind or rain; in that case – which happened only once –, it was moved to the next possible day. All monitoring was done by the same person. The resulting lists of detected birds per point for each of the five days were compiled in a species list. For a map with the exact site boundaries and point count locations see Figure 1.

### 2.3 Data analysis

To understand the changes in bird community compositions from 1850 to 2022, we assembled a species list with presence/absence data from each year based on the historic dataset and our own monitoring results from 2022. For a full bird species list with presence/absence data since 1850, see Table A1. Based on this compiled dataset, we plotted the species richness over the years and conducted a detrended correspondence analysis (DCA), a multivariate ordination technique used to explore and visualize patterns in species compositions (Hill & Gauch, 1980). We calculated a dissimilarity matrix of the bird communities using Jaccard's Index, an index suitable for handling sparse presence/absence data (Jaccard, 1902).

To investigate changes in guild occupations over the years, we assigned guilds to the species following the AVONET trait table by Tobias et al. (2022) for the traits habitat, primary lifestyle, trophic level, trophic niche, and migration. Each species is assigned to only one guild per trait. Based on the trait dataset, sankey graphs were produced that show the share of guild presence over the years. In addition, we calculated the functional richness using the five traits mentioned above.

All statistical analyses were done in R version 4.3.2 (R Core Team, 2023) with the help of packages vegan (Oksanen et al., 2022), ggplot2 (Wickham, 2016), tidyverse (Wickham et al., 2019), dplyr (Wickham et al., 2023), stringr (Wickham, 2023), readxl (Wickham & Bryan, 2023), wesanderson (Ram & Wickham, 2018), cowplot (Wilke, 2020), formattable (Ren & Russell, 2021), ggalluvial (Brunson & Read, 2023), mFD (Magneville et al., 2022), and gridExtra (Auguie, 2017).

## 3 RESULTS

### 3.1 Current bird community

In total, 54 different species were recorded in 2022. House Sparrow (*Passer domesticus*), Blackbird (*Turdus merula*), and Hooded Crow (*Corvus cornix*) accounted for the species with the highest occurrences, while the Green Woodpecker (*Picus viridis*), the Goldcrest (*Regulus regulus*), and the Firecrest (*Regulus ignicapilla*) were only recorded once. A full list of monitoring results including traits is in Table A1.

### 3.2 Changes in bird community composition over time

**FIGURE 2:**
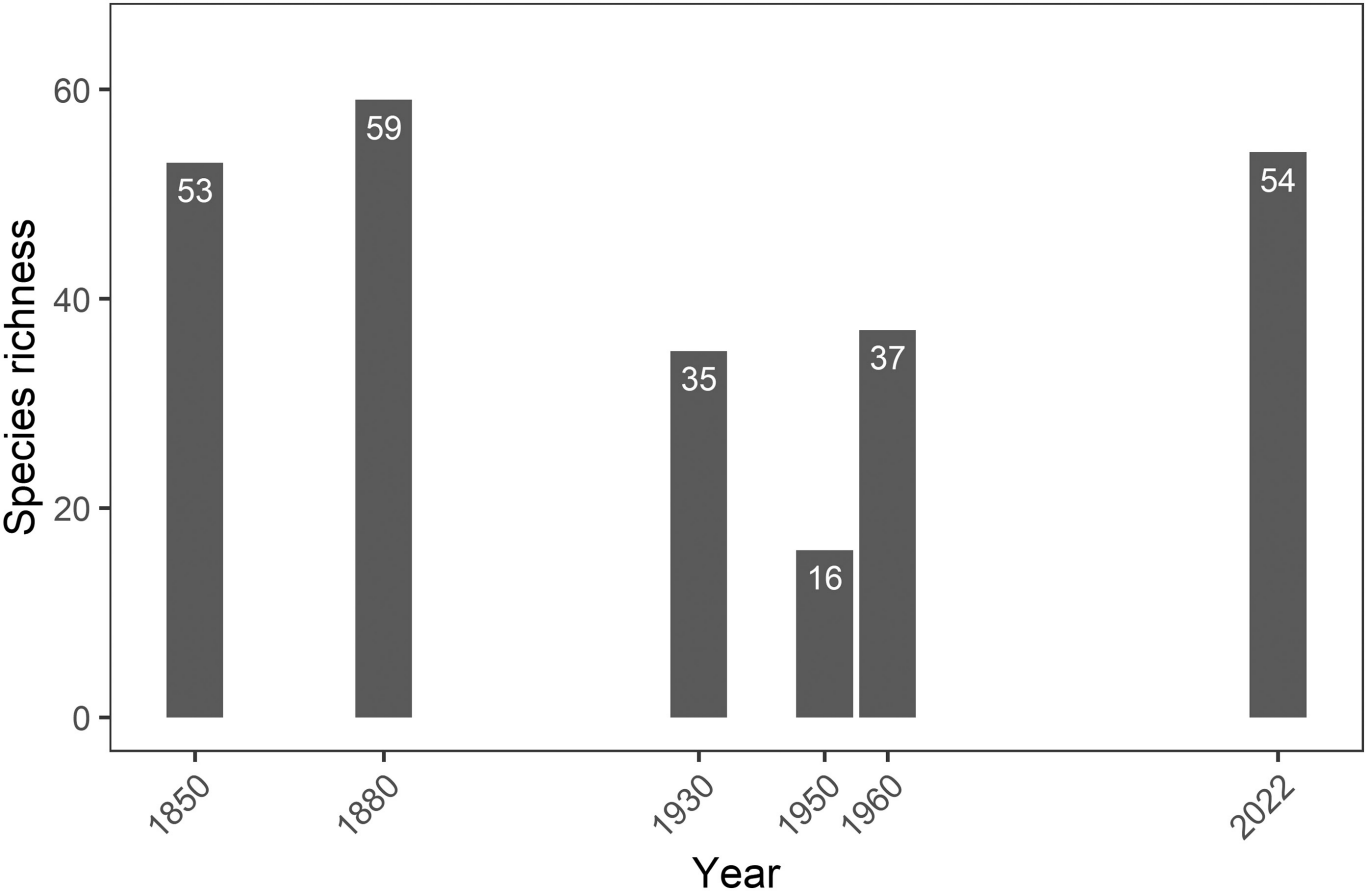
Species richness over the years in the Tiergarten in Berlin. Modified after Sprötge (1991) and complemented with own data for 2022.

The development of the species richness found in historical, recent, and current Tiergarten bird monitoring since 1850 is shown in Figure 2. The highest number of species (59) was found in 1880, and the least was found in 1950 (16). After 1950, the number increased again until reaching 54 species in 2022.

**FIGURE 3:**
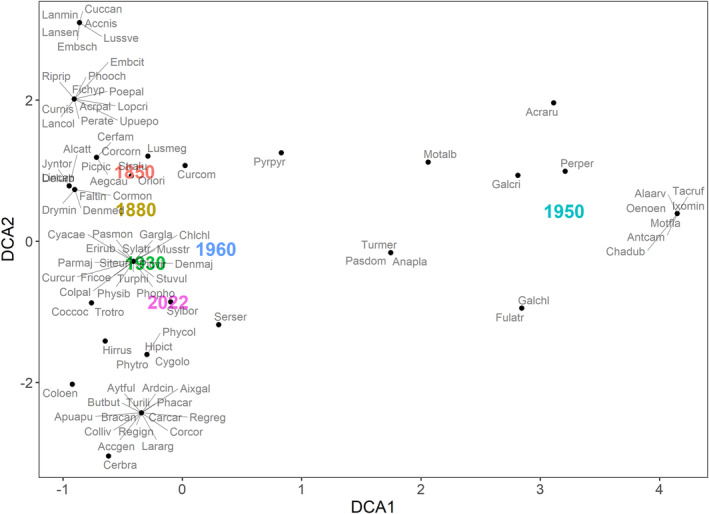
Detrended Correspondence Analysis (DCA) of the bird communities in Tiergarten between 1850 and 2022. Please see the Appendix for full species names. Calculation based on data between 1850 and 1960 as compiled by Sprötge (1991) and on data by the authors from 2022.

The species assemblages over the years are illustrated in the DCA (Figure 3). Most of the species communities of each year are in a row behind each other, with the smallest distance between the communities of 1930 and 1960. In contrast, the species community from 1950 is very far away from all the others. The species are grouped into a few clusters.

The dissimilarity matrix of the communities underlines the large differences between the community in 1950 and all other communities. It also shows how the assemblages from before 1950 are more similar to those after 1950 (see Table 1).

**TABLE 1:** Dissimilarity matrix between the bird communities in Tiergarten using Jaccard's dissimilarity index.

	1850	1880	1930	1950	1960
1880	0.30				
1930	0.51	0.44			
1950	0.92	0.93	0.94		
1960	0.62	0.50	0.40	0.83	
2022	0.63	0.55	0.54	0.91	0.48

*Note*: Columns and rows contain the years that represent the communities, as the species communities were recorded in those years.

### 3.3 Changes in guild occupation over time and functional richness

**FIGURE 4:**
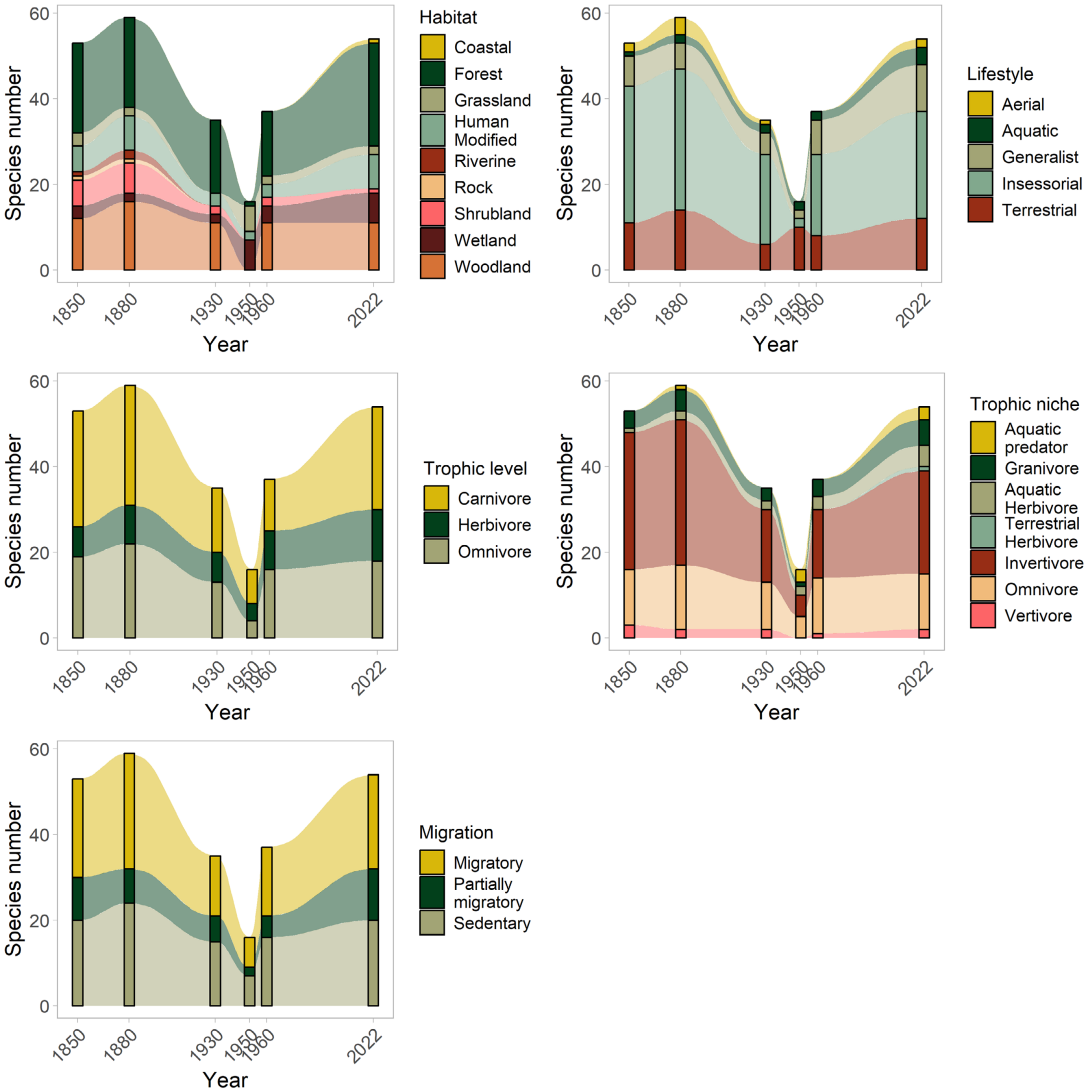
Absolute numbers of species occupying different guilds in historical and current bird communities in the Tiergarten in Berlin regarding habitat, lifestyle, trophic level, trophic niche, and migration. The bars with the darker colors show the presence as noted in the respective species lists per year. The sections with the paler colors in between show the presumed development in the years in between. Figure based on data from 1850 to 1960 by Sprötge (1991) and from 2022, as monitored by the authors. Guild attributions based on Tobias et al. (2022).

A visualization of the guild occupations of the traits habitat, lifestyle, trophic level, trophic niche, and migration is shown in Figure 4. Most species' habitats are forests or woodlands. Compared to 1850, riverine and rock species have disappeared in 2022, but one coastal species arrived. Over time, the share of generalist species increased, while the share of insessorial species decreased. Regarding the trophic level, the composition remained largely the same over time, with carnivores representing the biggest group, followed by omnivores and then by herbivores. When considering the trophic niches, the number of guilds increased from five to seven between 1850 and 2022 due to aquatic predators and aquatic herbivores. Both the absolute numbers and the share of the biggest group, the invertivores, decreased slightly. About the same number of species recorded in Tiergarten are migratory as sedentary, which also has not changed much over the years. See Table A2 in Appendix for a full list of species and trait assignments.

The functional richness over the years is shown in Table 2. Starting with 0.72 in 1850, it decreased until reaching 0.28 in 1950. Afterwards, it went up again, until reaching its highest point, 0.82, in 2022.

**TABLE 2:** Functional richness (Fric) of bird communities in the Tiergarten between 1850 and 2022.

	1850	1880	1930	1950	1960	2022
Fric	0.73	0.72	0.56	0.28	0.62	0.82

## 4 DISCUSSION

### 4.1 Bird community in 2022

The bird monitoring in 2022 resulted in 3091 observations of 54 species. They represent seven habitat guilds, five lifestyle guilds, three trophic levels, seven trophic niches, and three migratory statuses (see the detailed species lists in Appendix: Tables A1 and A2). That is a gratifyingly large number of species and more species than Scharon (2010) found in his monitoring in all five Berlin parks, although he mapped only breeding birds and omitted guests which we included. Nevertheless, this shows that a highly frequented park situated in a city center can harbor a high species richness. Of those species encountered in 2022, some are especially rare. The Common Moorhen, *Gallinula chloropus*, Barn Swallow, *Hirundo rustica*, and the Spotted Flycatcher *Muscicapa striata* are endangered in Berlin and Brandenburg. *Motacilla alba*, the White Wagtail, is classified as near‐threatened (Witt & Steiof, 2013). The two raptorial species, *Accipiter gentilis* (Northern Goshawk) and *Buteo buteo* (Common Buzzard), as well as the Common Moorhen and the Green Woodpecker, are strongly protected in Germany. Two species in Tiergarten are non‐native to the area: the Mandarin Duck, *Aix galericulata*, and Canada Goose, *Branta canadensis* (Witt & Steiof, 2013). The Mandarin Duck, which is native to China, was introduced to the Tiergarten in the 1920s and has since developed a stable breeding population. The Canada Goose was first sighted in Germany at the end of the 19th century and has since spread mainly to standing waters in urban regions (Bauer & Woog, 2008).

The presence of endangered and protected species underlines the Tiergarten's value in providing habitat for threatened species in an urban core. The large species numbers can be attributed to the park's size (i.e., Charre et al., 2013; Jokimäki 1999; Morelli et al., 2018; Schütz & Schulze, 2015), the presence of forest (Callaghan et al., 2020; Evans et al., 2018; Morelli et al., 2018; O'Neal, 2006; Planillo et al., 2021; Sandström et al., 2006), and the heterogeneity of the vegetation (Sandström et al., 2006; Sprötge, 1991). Furthermore, the presence of nest boxes supports certain species. Species that typically breed in nestboxes are the Great Tit, *Parus major*, the Blue Tit, *Cyanistes caerulaeus*, the House Sparrow, the Tree Sparrow *Passer montanus*, the Eurasian Nuthatch *Sitta europaea* and the Pied Flycatcher *Ficedula hypoleuca*. As Scharon (2020) notes, the high abundance of some of these species in the Tiergarten (especially Blue Tits, House Sparrows, and Great Tits) can be attributed to the plethora of nest boxes. To support these species in the future, it can be recommended to maintain these nextboxes. For a very detailed treatise on the exact relationships between different vegetation types in the Tiergarten and the bird species dependent on them, please refer to Sprötge (1991).

### 4.2 Urban exploiters, adapters and avoiders

*Apus apus* (Common Swift), House Sparrow, Hooded Crow, and *Sturnus vulgaris* (Common Starling) were among the species that were found in the highest abundance in 2022, a common result of bird monitoring in urban areas (see, for example, Fontana et al., 2011; Imai & Nakashizuka, 2010). These four species are typical urban exploiters (McKinney, 2002; Planillo et al., 2021). Planillo et al. (2021) conducted a joint species distribution model with the bird species they found in transects in Berlin (not only parks), which resulted in three clusters representing the three types of behavior towards urbanization. Their urban group, i.e., the urban exploiters, contains the Common Swift, the House Sparrow, the Hooded Crow, and the Common Starling, but also species such as the Blackbird, Rock Dove *Columba livia* and Wood Pigeon *Columba palumbus*. Their woodland birds or urban adapters group includes the Great and the Blue Tit, *Troglodytes troglodytes* (Wren), the European Nuthatch, *Fringilla coelebs* (Chaffinch), and *Erithacus rubecula* (Robin). Planillo et al. (2021)'s natural areas or urban avoiders group includes, among others, *Hippolais icterina* (Icterine warbler), *Serinus serinus* (Serin), *Silvia atricapilla* (Blackcap), *Luscinia megarhynchos* (Nightingale), *Carduelis carduelis* (Goldfinch), and *Carduelis chloris* (Greenfinch). These birds were also found in Tiergarten in our study in medium to high abundance. Consequently, the Tiergarten harbors species of all of these three groups, including those classified as urban avoiders by Planillo et al. (2021).

These findings in a single park demonstrate the Tiergarten's habitat quality for a variety of species with different requirements, which can be attributed to the park's heterogeneity and size.

### 4.3 Changes in bird and guild community composition over time

The changes in the number of species and also assemblages over the centuries are visible (Figures 2 and 3 and Table 1). Most striking is the uniqueness of the 1950s community. The communities from 1930 and 1960 are much more similar to each other than they are to the 1950s. Furthermore, a gradual change in species composition over the years is demonstrated by lining up communities in chronic order in the plot. Thus, except for the sharp divergence of the 1950 community, there have been gradual progressive changes since 1850.

The development of the historical landscape structure, which Sprötge (1991) researched in detail, can be traced through the guilds (see Figure 4). The Tiergarten was redesigned into a park landscape with a high proportion of forest in the first half of the 19th century. In 1850, during the first bird mapping, most species had their habitats in forests or woodlands, but there were also shrubland and grassland species present such as the Hoopoe *Upupa epops*, the Golden Oriole *Oriolus oriolus*, the Bluethroat *Luscinia svecica*, or the Yellowhammer *Emberiza citrinella* as well as a few wetland species – possibly due to the high structural richness as well as remnants of swamp forests caused by the close‐by river Spree. Most species were insessorial, reflecting the vertical vegetation structure. Sprötge (1991) attributed the general species decline in 1930 to the fact that woody stands were becoming denser and, as a result, the herbaceous and shrub layers were degenerating. He quotes an anonymous author who writes about a paucity of songbirds in the Tiergarten around 1900 and also explains the simultaneous decline in breeding waterfowl by the fact that there was hardly any riparian vegetation left due to the heavy shading by the trees. Looking at the habitat diagram in Figure 4, we see a decrease in riverine, grassland, and shrubland species.

After World War II, the Tiergarten was almost completely deforested and most of the previously present species disappeared – only *Anas platyrhynchos* (Mallard), House Sparrow and Blackbird remained and are thus the only three species that have been breeding in the Tiergarten since records began. A community of open land species formed on the debris and fields, including the Skylark *Alauda arvensis*, the Tawny Pipit *Anthus campestris*, the Yellow Wagtail *Motacilla flava*, the Northern Wheatear *Oenanthe oenanthe*, and *Perdix perdix*, the Gray Partridge. The riparian vegetation, now well developed, also favored the occurrence of reed species like *Ixobrychus minutus* (Little bittern), *Charadrius dubius* (Little ringed plover), and *Tachybaptus ruficollis* (Little grebe) (Sprötge, 1991).

The decrease in species richness explains most of the variation in guild occupation visible in 1950: the drop is reflected in a decrease in almost all habitat guilds except for wetland and grassland species, and almost all feeding guilds except for aquatic predators and aquatic herbivores. Some of the insessorial species likewise disappeared, while terrestrial species increased. It should, however, be noted that the small species number recorded in 1950 could also be due to the dramatic consequences of the war, which potentially influenced the time spent bird‐watching.

Reforestation since 1949 had displaced the open land species again, and forest, woodland, and shrub species returned or re‐established instead (Scharon, 2015; Sprötge, 1991). This process is reflected by an increase in insessorial and invertivore species. The development towards a park bird community, including species populating settlements or forests, continues to progress since then (Scharon, 2020). In times of tighter budgets for park maintenance and increasing pressure on use (Haaland & van den Bosch, 2015), it is to be hoped that the Tiergarten will be able to maintain its diversity and that there will not be a similar development as in the 1930s when many species disappeared due to a lack of forest thinning which impaired the shrub layer (Sprötge, 1991). However, the increase in the number of species and the (re‐)introduction of several species does corroborate the sound state of the Tiergarten as a habitat providing for the different needs of its bird community. It can therefore be recommended to allocate sufficient funding for park maintenance to ensure necessary interventions.

### 4.4 Guild occupation in the context of urbanization

The often‐reported functional homogenization as a result of urbanization cannot be confirmed for Tiergarten. Despite some small changes, guild occupations for the traits considered in this study in 2022 are similar to those of 1850.

The park harbors a high proportion of invertivores, a medium proportion of omnivores, and a low proportion of granivores. Many studies found evidence for the assumption that urbanization selects omnivores and granivores and harbors only low numbers of invertivores (i.e., Callaghan et al., 2019; Chace & Walsh, 2006; Croci et al., 2008; Devictor et al., 2007; Imai & Nakashizuka, 2010). Omnivores and, in general, species with a wider diet profit from urbanization as they can take advantage of garbage as a food source (Clergeau et al., 1998), while invertivores and, in general, species with narrower diets have difficulties finding food (Palacio, 2020; Patankar et al., 2021). This cannot be confirmed for the Tiergarten, probably because the Tiergarten does not have the typical features of highly urbanized landscapes.

The majority of studies that found primarily omnivores examined transects in urban regions (Callaghan et al., 2019; Croci et al., 2008; Devictor et al., 2007) or compared small parks of mostly less than 10 ha (Imai & Nakashizuka, 2010). These effects of urbanization cannot be demonstrated for the Tiergarten, which seems to mediate them due to its size and properties. A study of many different habitats in Europe (not just urban) shows that the majority of European breeding birds are indeed invertivores (Bowler et al., 2019). The authors of this study also found a sharp decline in insectivores over 25 years, which cannot be confirmed for the Tiergarten either. Even considering the trends in the proportions of feeding guilds, the share of omnivores in 2022 is similar to that of 1850.

Scharon (2020) associates the decline in the bush breeding species *Pica pica* (Magpie), *Chloris chloris* (Greenfinch), *Sylvia borin* (Garden Warbler), and *Sylvia curruca* (Lesser Whitethroat) from 1988 to 2010 with a decrease in understory vegetation due to the growth of young trees, a trend that seems to have continued until today. In turn, these trees, now advanced in age and size, have probably favored the population of Hooded Crows (Scharon, 2020).

Remarkably, the occupation of guilds has changed only marginally over the centuries despite changes in species composition. Species turnover over time is not surprising and is often characterized by loss of species richness and dominance of a few species as urbanization progresses (Chace & Walsh, 2006; Palacio et al., 2018; Sol et al., 2020). However, the often‐proclaimed biotic homogenization, i.e., the decrease of functional diversity in urban areas (Devictor et al., 2007; La Sorte et al., 2018; Marzluff, 2017) could not be proven for Tiergarten for the investigated traits during the last 172 years: despite intensifying urbanization the functional richness in 2022 was higher than ever before. Indeed, some studies corroborate our findings. Sol et al. (2020) detected a reduced functional diversity in cities compared to surrounding natural areas due to a decrease in species richness and abundance which resulted in a loss of functional redundancy, i.e., fewer species covering similar traits. Their data contradict the hypothesis that functionally unique species are less tolerant to urbanization. In a meta‐study on urban bird assemblages from 25 cities across the globe, Oliveira Hagen et al. (2017) found evidence for higher functional diversity in cities compared to nonurban surroundings, which they attributed to higher habitat heterogeneity in cities. Although we did not compare our results with a non‐urban location and the Tiergarten was already an inner‐city park in 1850, it can be stated that the ongoing urbanization since then has still not resulted in a significant decline in guild occupation. It should be noted, however, that the size of the park is most likely largely responsible for this diversity – although this is all the more impressive given the fact that the park is bisected by several busy roads. Our results thus provide proof that cities can support diverse avian communities without detectable functional homogenization based on a long‐term study dataset. We recommend conducting further research on guild occupations in urban assemblages and biotic homogenization to gain a better understanding of relationships between urban features and functional diversity.

## 5 CONCLUSION

In this study, insights into the taxonomic and functional diversity of current and historical avian communities in the Berlin Tiergarten could be gained. It therefore, constitutes one of the rare long‐term studies on the effects of urbanization on bird diversity. Among the current species were those classified as urban exploiters, adapters, and avoiders. This points out the park's value as a habitat while demonstrating that an intense use by humans nearly around the clock can still support high bird diversity.

No sign of functional homogenization could be found for the traits habitat, primary lifestyle, trophic level, trophic niche, and migration, which underlines the possibility of a park maintaining considerably high bird (functional) diversity despite being highly frequented and situated right in a city center.

In general, the Berlin Tiergarten is of high value for Berlin's residents, tourists, and many species. Two central assets for both humans and animals are its remarkable size and its heterogeneity. It can be recommended to maintain both to allow a continued co‐existence of manifold users and inhabitants of the park in the future. The bird communities in Tiergarten follow patterns over time that are not usually associated with urban centers. We advise maintaining large sizes and high habitat heterogeneity with structured vegetation layers in urban parks, i.e., not only incorporate water bodies and open areas but also forested parts with understory vegetation to ensure habitat quality for a variety of species and guilds.

## AUTHOR CONTRIBUTIONS

**Nadja Pernat:** Visualization (supporting); writing – review and editing (equal). **Esther Sophie Felgentreff:** Conceptualization (lead); data curation (lead); formal analysis (lead); methodology (lead); visualization (lead); writing – original draft (lead); writing – review and editing (lead). **Sascha Buchholz:** Formal analysis (supporting); supervision (lead); writing – review and editing (equal).

## CONFLICT OF INTEREST STATEMENT

All authors confirm that they do not have any competing interests.

## Data Availability

All of our data is presented in Tables A1 and A2 in Appendix.

## Appendix

**TABLE A1:** Species in Tiergarten between 1850 and 2022.

Species	Common name	Abbreviation	1850	1880	1930	1950	2022
*Accipiter gentilis*	Northern goshawk	Accgen	0	0	0	0	1
*Accipiter nisus*	Sparrowhawk	Accnis	1	0	0	0	0
*Acrocephalus arundinaceus*	Great reed warbler	Acraru	1	0	0	1	0
*Acrocephalus palustris*	Marsh warbler	Acrpal	1	1	0	0	0
*Aegithalos caudatus*	Long‐tailed tit	Aegcau	1	1	0	0	1
*Aix galericulata*	Mandarin duck	Aixgal	0	0	0	0	1
*Alauda arvensis*	Skylark	Alaarv	0	0	0	1	0
*Alcedo atthis*	Kingfisher	Alcatt	0	1	0	0	0
*Anas platyrhynchos*	Mallard	Anapla	1	1	1	1	1
*Anthus campestris*	Tawny pipit	Antcam	0	0	0	1	0
*Apus apus*	Common swift	Apuapu	0	0	0	0	1
*Ardea cinerea*	Gray heron	Ardcin	0	0	0	0	1
*Aythya fuligula*	Tufted duck	Aytful	0	0	0	0	1
*Branta canadensis*	Canada goose	Bracan	0	0	0	0	1
*Buteo buteo*	Common buzzard	Butbut	0	0	0	0	1
*Carduelis carduelis*	Goldfinch	Carcar	0	0	0	0	1
*Certhia brachydactyla*	Short‐toed treecreeper	Cerbra	0	0	1	0	1
*Certhia familiaris*	Eurasian treecreeper	Cerfam	1	1	0	0	1
*Charadrius dubius*	Little ringed plover	Chadub	0	0	0	1	0
*Chloris chloris*	Greenfinch	Chlchl	1	1	1	0	1
*Coccothraustes coccothraustes*	Hawfinch	Coccoc	1	1	1	0	1
*Columba livia*	Rock dove	Colliv	0	0	0	0	1
*Columba oenas*	Stock dove	Coloen	0	1	1	0	0
*Columba palumbus*	Wood pigeon	Colpal	1	1	1	0	1
*Corvus corax*	Common raven	Corcor	0	0	0	0	1
*Corvus cornix*	Hooded crow	Corcorn	1	1	0	0	1
*Corvus monedula*	Western jackdaw	Cormon	1	1	1	0	0
*Cuculus canorus*	Cuckoo	Cuccan	1	0	0	0	0
*Curruca communis*	Common whitethroat	Curcom	0	1	0	0	0
*Curruca curruca*	Lesser whitethroat	Curcur	1	1	1	0	1
*Curruca nisoria*	Barred warbler	Curnis	1	1	0	0	0
*Cyanistes caeruleus*	Blue tit	Cyacae	1	1	1	0	1
*Cygnus olor*	Mute swan	Cygolo	0	1	1	0	1
*Delichon urbicum*	House martin	Delurb	0	1	0	0	0
*Dendrocopos major*	Great spotted woodpecker	Denmaj	1	1	1	0	1
*Dendrocoptes medius*	Middle spotted woodpecker	Denmed	1	1	1	0	0
*Dryobates minor*	Lesser spotted woodpecker	Drymin	1	1	1	0	0
*Emberiza citrinella*	Yellowhammer	Embcit	1	1	0	0	0
*Emberiza schoeniclus*	Reed bunting	Embsch	1	0	0	0	0
*Erithacus rubecula*	Robin	Erirub	1	1	1	0	1
*Falco tinnunculus*	Kestrel	Faltin	1	1	1	0	0
*Ficedula hypoleuca*	Pied flycatcher	Fichyp	1	1	0	0	0
*Fringilla coelebs*	Chaffinch	Fricoe	1	1	1	0	1
*Fulica atra*	Coot	Fulatr	0	0	0	1	1
*Galerida cristata*	Crested lark	Galcri	0	1	0	1	0
*Gallinula chloropus*	Moorhen	Galchl	0	0	0	1	1
*Garrulus glandarius*	Jay	Gargla	1	1	1	0	1
*Hippolais icterina*	Icterine warbler	Hipict	0	1	1	0	1
*Hirundo rustica*	Barn swallow	Hirrus	0	1	0	0	1
*Ixobrychus minutus*	Little bittern	Ixomin	0	0	0	1	0
*Jynx torquilla*	Wryneck	Jyntor	0	1	0	0	0
*Lanius collurio*	Red‐backed shrike	Lancol	1	1	0	0	0
*Lanius minor*	Lesser gray shrike	Lanmin	1	0	0	0	0
*Lanius senator*	Woodchat shrike	Lansen	1	0	0	0	0
*Larus argentatus*	Herring gull	Lararg	0	0	0	0	1
*Linaria cannabina*	Common linnet	Lincan	0	1	0	0	0
*Lophophanes cristatus*	Crested tit	Lopcri	1	1	0	0	0
*Luscinia megarhynchos*	Nightingale	Lusmeg	1	1	0	0	1
*Luscinia svecica*	Bluethroat	Lussve	1	0	0	0	0
*Motacilla alba*	Pied wagtail	Motalb	1	1	0	1	1
*Motacilla flava*	Yellow wagtail	Motfla	0	0	0	1	0
*Muscicapa striata*	Spotted flycatcher	Musstr	1	1	1	0	1
*Oenanthe oenanthe*	Wheatear	Oenoen	0	0	0	1	0
*Oriolus oriolus*	Oriole	Oriori	1	1	1	0	0
*Parus major*	Great tit	Parmaj	1	1	1	0	1
*Passer domesticus*	House sparrow	Pasdom	1	1	1	1	1
*Passer montanus*	Tree sparrow	Pasmon	1	1	1	0	1
*Perdix perdix*	Gray partridge	Perper	0	0	0	1	0
*Periparus ater*	Coal tit	Perate	1	1	0	0	0
*Phalacrocorax carbo*	Cormorant	Phacar	0	0	0	0	1
*Phoenicurus ochruros*	Black redstart	Phooch	1	1	0	0	0
*Phoenicurus phoenicurus*	Common redstart	Phopho	1	1	1	0	1
*Phylloscopus collybita*	Common chiffchaff	Phycol	0	1	1	0	1
*Phylloscopus sibilatrix*	Wood warbler	Physib	1	1	1	0	1
*Phylloscopus trochilus*	Willow warbler	Phytro	0	1	1	0	1
*Pica pica*	Common magpie	Picpic	1	1	0	0	1
*Picus viridis*	Green woodpecker	Picvir	1	1	1	0	1
*Poecile palustris*	Marsh tit	Poepal	1	1	0	0	0
*Pyrrhula pyrrhula*	Common bullfinch	Pyrpyr	0	0	0	0	0
*Regulus ignicapilla*	Common firecrest	Regign	0	0	0	0	1
*Regulus regulus*	Goldcrest	Regreg	0	0	0	0	1
*Riparia riparia*	Sand martin	Riprip	1	1	0	0	0
*Serinus serinus*	Serin	Serser	0	0	0	0	1
*Sitta europaea*	Eurasian nuthatch	Siteur	1	1	1	0	1
*Strix aluco*	Tawny owl	Stralu	1	1	1	0	0
*Sturnus vulgaris*	Common starling	Stuvul	1	1	1	0	1
*Sylvia atricapilla*	Blackcap	Sylatr	1	1	1	0	1
*Sylvia borin*	Garden warbler	Sylbor	0	1	0	0	1
*Tachybaptus ruficollis*	Little grebe	Tacruf	0	0	0	1	0
*Troglodytes troglodytes*	Wren	Trotro	1	1	1	0	1
*Turdus iliacus*	Redwing	Turili	0	0	0	0	1
*Turdus merula*	Blackbird	Turmer	1	1	1	1	1
*Turdus philomelos*	Song thrush	Turphi	1	1	1	0	1
*Upupa epops*	Hoopoe	Upuepo	1	1	0	0	0

*Note*: 1 means a species was recorded in the respective year, 0 means it was not. Data from 1850 to 1960 compiled by Sprötge (1991), and from 2022 as monitored by the authors. Species lists contain all bird species recorded in Tiergarten including guests.

**TABLE A2:** Trait assignments of the species found in Tiergarten between 1850 and 2022 based on the AVONET trait table by Tobias et al. (2022).

Species	Habitat	Primary lifestyle	Trophic level	Trophic niche	Migration
*Accipiter gentilis*	Forest	Generalist	Carnivore	Vertivore	Partially migratory
*Accipiter nisus*	Forest	Insessorial	Carnivore	Vertivore	Partially migratory
*Acrocephalus arundinaceus*	Wetland	Insessorial	Carnivore	Invertivore	Migratory
*Acrocephalus palustris*	Shrubland	Insessorial	Carnivore	Invertivore	Migratory
*Aegithalos caudatus*	Forest	Insessorial	Carnivore	Invertivore	Sedentary
*Aix galericulata*	Forest	Generalist	Herbivore	Herbivore aquatic	Migratory
*Alauda arvensis*	Grassland	Terrestrial	Omnivore	Omnivore	Partially migratory
*Alcedo atthis*	Riverine	Insessorial	Carnivore	Aquatic predator	Sedentary
*Anas platyrhynchos*	Wetland	Aquatic	Herbivore	Herbivore aquatic	Partially migratory
*Anthus campestris*	Grassland	Terrestrial	Carnivore	Invertivore	Migratory
*Apus apus*	Human modified	Aerial	Carnivore	Invertivore	Migratory
*Ardea cinerea*	Wetland	Terrestrial	Carnivore	Aquatic predator	Sedentary
*Aythya fuligula*	Wetland	Aquatic	Omnivore	Herbivore aquatic	Partially migratory
*Branta canadensis*	Grassland	Terrestrial	Herbivore	Herbivore terrestrial	Migratory
*Buteo buteo*	Grassland	Insessorial	Carnivore	Vertivore	Partially migratory
*Carduelis carduelis*	Woodland	Insessorial	Herbivore	Granivore	Sedentary
*Certhia brachydactyla*	Forest	Insessorial	Carnivore	Invertivore	Sedentary
*Certhia familiaris*	Forest	Insessorial	Omnivore	Invertivore	Sedentary
*Charadrius dubius*	Wetland	Terrestrial	Carnivore	Aquatic predator	Migratory
*Chloris chloris*	Woodland	Generalist	Herbivore	Granivore	Sedentary
*Coccothraustes coccothraustes*	Forest	Insessorial	Herbivore	Omnivore	Partially migratory
*Columba livia*	Human modified	Terrestrial	Herbivore	Granivore	Sedentary
*Columba oenas*	Woodland	Terrestrial	Herbivore	Omnivore	Sedentary
*Columba palumbus*	Woodland	Terrestrial	Herbivore	Omnivore	Sedentary
*Corvus corax*	Forest	Terrestrial	Omnivore	Omnivore	Partially migratory
*Corvus cornix*	Human modified	Terrestrial	Carnivore	Omnivore	Sedentary
*Corvus monedula*	Human modified	Terrestrial	Omnivore	Omnivore	Partially migratory
*Cuculus canorus*	Forest	Insessorial	Carnivore	Invertivore	Migratory
*Curruca communis*	Shrubland	Insessorial	Omnivore	Invertivore	Migratory
*Curruca curruca*	Shrubland	Insessorial	Omnivore	Invertivore	Migratory
*Curruca nisoria*	Shrubland	Insessorial	Carnivore	Invertivore	Migratory
*Cyanistes caeruleus*	Forest	Insessorial	Omnivore	Invertivore	Sedentary
*Cygnus olor*	Wetland	Aquatic	Herbivore	Herbivore aquatic	Migratory
*Delichon urbicum*	Human modified	Aerial	Carnivore	Invertivore	Migratory
*Dendrocopos major*	Woodland	Insessorial	Omnivore	Omnivore	Sedentary
*Dendrocoptes medius*	Woodland	Insessorial	Carnivore	Invertivore	Sedentary
*Dryobates minor*	Forest	Insessorial	Carnivore	Invertivore	Sedentary
*Emberiza citrinella*	Shrubland	Terrestrial	Herbivore	Granivore	Partially migratory
*Emberiza schoeniclus*	Wetland	Terrestrial	Herbivore	Omnivore	Partially migratory
*Erithacus rubecula*	Forest	Generalist	Carnivore	Omnivore	Migratory
*Falco tinnunculus*	Shrubland	Aerial	Carnivore	Vertivore	Migratory
*Ficedula hypoleuca*	Forest	Insessorial	Carnivore	Invertivore	Migratory
*Fringilla coelebs*	Forest	Generalist	Omnivore	Invertivore	Partially migratory
*Fulica atra*	Wetland	Generalist	Herbivore	Herbivore aquatic	Sedentary
*Galerida cristata*	Grassland	Terrestrial	Omnivore	Omnivore	Sedentary
*Gallinula chloropus*	Wetland	Terrestrial	Omnivore	Omnivore	Sedentary
*Garrulus glandarius*	Forest	Insessorial	Omnivore	Omnivore	Partially migratory
*Hippolais icterina*	Woodland	Insessorial	Carnivore	Invertivore	Migratory
*Hirundo rustica*	Human modified	Aerial	Carnivore	Invertivore	Migratory
*Ixobrychus minutus*	Wetland	Terrestrial	Carnivore	Aquatic predator	Migratory
*Jynx torquilla*	Woodland	Terrestrial	Carnivore	Invertivore	Migratory
*Lanius collurio*	Woodland	Insessorial	Carnivore	Invertivore	Migratory
*Lanius minor*	Grassland	Insessorial	Carnivore	Invertivore	Migratory
*Lanius senator*	Shrubland	Insessorial	Carnivore	Invertivore	Migratory
*Larus argentatus*	Coastal	Generalist	Carnivore	Aquatic predator	Partially migratory
*Linaria cannabina*	Shrubland	Terrestrial	Herbivore	Granivore	Sedentary
*Lophophanes cristatus*	Woodland	Insessorial	Omnivore	Invertivore	Sedentary
*Luscinia megarhynchos*	Woodland	Generalist	Carnivore	Invertivore	Migratory
*Luscinia svecica*	Grassland	Generalist	Carnivore	Invertivore	Migratory
*Motacilla alba*	Human modified	Terrestrial	Carnivore	Invertivore	Migratory
*Motacilla flava*	Grassland	Terrestrial	Carnivore	Invertivore	Migratory
*Muscicapa striata*	Forest	Insessorial	Carnivore	Invertivore	Migratory
*Oenanthe oenanthe*	Grassland	Insessorial	Carnivore	Invertivore	Migratory
*Oriolus oriolus*	Woodland	Insessorial	Omnivore	Omnivore	Migratory
*Parus major*	Woodland	Insessorial	Omnivore	Invertivore	Sedentary
*Passer domesticus*	Human modified	Terrestrial	Herbivore	Granivore	Sedentary
*Passer montanus*	Woodland	Terrestrial	Omnivore	Granivore	Sedentary
*Perdix perdix*	Grassland	Terrestrial	Herbivore	Omnivore	Sedentary
*Periparus ater*	Forest	Insessorial	Omnivore	Invertivore	Sedentary
*Phalacrocorax carbo*	Wetland	Aquatic	Carnivore	Aquatic predator	Partially migratory
*Phoenicurus ochruros*	Rock	Insessorial	Omnivore	Invertivore	Partially migratory
*Phoenicurus phoenicurus*	Woodland	Insessorial	Carnivore	Invertivore	Migratory
*Phylloscopus collybita*	Forest	Insessorial	Carnivore	Invertivore	Migratory
*Phylloscopus sibilatrix*	Forest	Insessorial	Carnivore	Invertivore	Migratory
*Phylloscopus trochilus*	Forest	Insessorial	Carnivore	Invertivore	Migratory
*Pica pica*	Human modified	Terrestrial	Omnivore	Omnivore	Sedentary
*Picus viridis*	Forest	Terrestrial	Carnivore	Invertivore	Sedentary
*Poecile palustris*	Forest	Insessorial	Omnivore	Invertivore	Sedentary
*Pyrrhula pyrrhula*	Forest	Insessorial	Herbivore	Omnivore	Sedentary
*Regulus ignicapilla*	Forest	Insessorial	Carnivore	Invertivore	Migratory
*Regulus regulus*	Forest	Insessorial	Carnivore	Invertivore	Migratory
*Riparia riparia*	Riverine	Aerial	Carnivore	Invertivore	Migratory
*Serinus serinus*	Forest	Generalist	Herbivore	Granivore	Partially migratory
*Sitta europaea*	Forest	Insessorial	Carnivore	Invertivore	Sedentary
*Strix aluco*	Forest	Insessorial	Carnivore	Vertivore	Sedentary
*Sturnus vulgaris*	Human modified	Insessorial	Omnivore	Omnivore	Partially migratory
*Sylvia atricapilla*	Woodland	Insessorial	Omnivore	Omnivore	Migratory
*Sylvia borin*	Woodland	Insessorial	Omnivore	Omnivore	Migratory
*Tachybaptus ruficollis*	Wetland	Aquatic	Carnivore	Aquatic predator	Sedentary
*Troglodytes troglodytes*	Forest	Insessorial	Carnivore	Invertivore	Migratory
*Turdus iliacus*	Forest	Insessorial	Omnivore	Invertivore	Migratory
*Turdus merula*	Forest	Generalist	Omnivore	Omnivore	Sedentary
*Turdus philomelos*	Forest	Generalist	Omnivore	Invertivore	Migratory
*Upupa epops*	Grassland	Terrestrial	Carnivore	Invertivore	Migratory

## References

[ece311461-bib-0001] Amt für Statistik Berlin Brandenburg . (2023). *Einwohnerinnen und Einwohner im Land Berlin am 30 Juni 2023*. [Internet]. https://www.statistik‐berlin‐brandenburg.de/a‐i‐7‐a‐ii‐3‐a‐iii‐3‐m

[ece311461-bib-0002] Amt für Statistik Berlin‐Brandenburg . (2019). *Flächennutzung/Gebiet* [Internet]. https://www.statistik‐berlin‐brandenburg.de/BasisZeitreiheGrafik/Bas‐Flaechennutzung.asp?Ptyp=300&Sageb=33000&creg=BBB&anzwer=6

[ece311461-bib-0004] Aronson, M. F. , La Sorte, F. A. , Nilon, C. H. , Katti, M. , Goddard, M. A. , Lepczyk, C. A. , Warren, P. S. , Williams, N. S. , Cilliers, S. , Clarkson, B. , Dobbs, C. , Dolan, R. , Hedblom, M. , Klotz, S. , Kooijmans, J. L. , Kühn, I. , Macgregor‐Fors, I. , McDonnell, M. , Mörtberg, U. , … Winter, M. (2014). A global analysis of the impacts of urbanization on bird and plant diversity reveals key anthropogenic drivers. Proceedings of the Royal Society B, 281(1780), 20133330. 10.1098/rspb.2013.3330 24523278 PMC4027400

[ece311461-bib-0005] Auguie, B. (2017). *gridExtra: Miscellaneous functions for “grid” graphics* [Internet]. https://CRAN.R‐project.org/package=gridExtra

[ece311461-bib-0006] Bauer, H. G. , & Woog, F. (2008). Nichtheimische Vogelarten (Neozoen) in Deutschland, Teil I: Auftreten, Bestände und Status. Vogelwarte, 46, 157–194.

[ece311461-bib-0007] Bibby, C. J. , Burgess, N. D. , & Hill, D. A. (1995). Punkt‐Stopp‐Zählungen (Punkttaxierungen). In C. J. Bibby , N. D. Burgess , & D. Hill (Eds.), Methoden der Feldornithologie – Bestandserfassung in der Praxis (pp. 99–118). Neumann.

[ece311461-bib-0009] Bowler, D. E. , Heldbjerg, H. , Fox, A. D. , Jong, M. , & Böhning‐Gaese, K. (2019). Long‐term declines of European insectivorous bird populations and potential causes. Conservation Biology, 33(5), 1120–1130. 10.1111/cobi.13307 30912605

[ece311461-bib-0010] Brunson, J. C. , & Read, Q. (2023). *ggalluvial: Alluvial plots in “ggplot2”* [Internet]. http://corybrunson.github.io/ggalluvial/

[ece311461-bib-0011] Callaghan, C. T. , Major, R. E. , Wilshire, J. H. , Martin, J. M. , Kingsford, R. T. , & Cornwell, W. K. (2019). Generalists are the most urban‐tolerant of birds: A phylogenetically controlled analysis of ecological and life history traits using a novel continuous measure of bird responses to urbanization. Oikos, 128(6), 845–858. 10.1111/oik.06158

[ece311461-bib-0012] Callaghan, C. T. , Palacio, F. X. , Benedetti, Y. , Morelli, F. , & Bowler, D. E. (2023). Large‐scale spatial variability in urban tolerance of birds. Journal of Animal Ecology, 92(2), 403–416. 36477754 10.1111/1365-2656.13862

[ece311461-bib-0013] Callaghan, C. T. , Poore, A. G. B. , Major, R. E. , Cornwell, W. K. , Wilshire, J. H. , & Lyons, M. B. (2020). How to build a biodiverse city: Environmental determinants of bird diversity within and among 1581 cities. Biodiversity and Conservation, 30(1), 217–234.

[ece311461-bib-0015] Chace, J. F. , & Walsh, J. J. (2006). Urban effects on native avifauna: A review. Landscape and Urban Planning, 74(1), 46–69. 10.1016/j.landurbplan.2004.08.007

[ece311461-bib-0016] Charre, G. M. , Hurtado, J. A. Z. , Néve, G. , Ponce‐Mendoza, A. , & Corcuera, P. (2013). Relationship between habitat traits and bird diversity and composition in selected urban green areas of Mexico City. Ornitologia Neotropical, 24, 275–293.

[ece311461-bib-0017] Clergeau, P. , Savard, J. P. L. , Mennechez, G. , & Falardeau, G. (1998). Bird abundance and diversity along an urban‐rural gradient: A comparative study between two cities on different continents. The Condor, 100(3), 413–425. 10.2307/1369707

[ece311461-bib-0018] Croci, S. , Butet, A. , & Clergeau, P. (2008). Does urbanization filter birds on the basis of their biological traits? Condor, 110(2), 223–240. 10.1525/cond.2008.8409

[ece311461-bib-0019] Devictor, V. , Julliard, R. , Couvet, D. , Lee, A. , & Jiguet, F. (2007). Functional homogenization effect of urbanization on bird communities. Conservation Biology, 21(3), 741–751. 10.1111/j.1523-1739.2007.00671.x 17531052

[ece311461-bib-0020] DWD . (2013a). *Temperatur: Langjährige Mittelwerte 1981–2010, Bezugsstandort: Berlin‐Dahlem* [Internet]. Deutscher Wetterdienst. http://archive.ph/Y5TWm

[ece311461-bib-0021] DWD . (2013b). *Niederschlag: langjährige Mittelwerte 1981–2010, Bezugsstandort: Berlin‐Dahlem* [Internet]. Deutscher Wetterdienst. http://archive.ph/Y5TWm

[ece311461-bib-0022] EEA . (2020). Environmental noise in Europe – 2020 [Internet] (p. 104). European Environment Agency. Report No.: 22/2019. https://www.eea.europa.eu/publications/environmental‐noise‐in‐europe

[ece311461-bib-0023] Evans, B. S. , Reitsma, R. , Hurlbert, A. H. , & Marra, P. P. (2018). Environmental filtering of avian communities along a rural‐to‐urban gradient in Greater Washington, DC, USA. Ecosphere, 9(11), 1–19. 10.1002/ecs2.2402 38357012

[ece311461-bib-0024] Faeth, S. H. , Saari, S. , & Bang, C. (2012). Urban biodiversity: Patterns, processes and implications for conservation. In eLS [Internet] (pp. 1–12). John Wiley & Sons, Ltd. 10.1002/9780470015902.a0023572

[ece311461-bib-0025] Fischer, S. , Flade, M. , & Schwarz, J. (2005). Punkt‐Stopp‐Zählung. In P. Südbeck , H. Andretzke , S. Fischer , K. Gedeon , T. Schikore , K. Schröder , & C. Sudfeldt (Eds.), Methodenstandards zur Erfassung der Brutvögel Deutschlands (pp. 54–58). Max‐Planck‐Institut für Ornithologie.

[ece311461-bib-0026] Fontana, S. , Sattler, T. , Bontadina, F. , & Moretti, M. (2011). How to manage the urban green to improve bird diversity and community structure. Landscape and Urban Planning, 101(3), 278–285. 10.1016/j.landurbplan.2011.02.033

[ece311461-bib-0027] Gagné, S. A. , Sherman, P. J. , Singh, K. K. , & Meentemeyer, R. K. (2016). The effect of human population size on the breeding bird diversity of urban regions. Biodiversity and Conservation, 25(4), 653–671. 10.1007/s10531-016-1080-3

[ece311461-bib-0028] Google Earth . (2020). Map of Berlin .

[ece311461-bib-0029] Grimm, N. B. , Faeth, S. H. , Golubiewski, N. E. , Redman, C. L. , Wu, J. , Bai, X. , & Briggs, J. M. (2008). Global change and the ecology of cities. Science, 319(5864), 756–760. 10.1126/science.1150195 18258902

[ece311461-bib-0030] Haaland, C. , & van den Bosch, C. K. (2015). Challenges and strategies for urban green‐space planning in cities undergoing densification: A review. Urban Forestry & Urban Greening, 14(4), 760–771. 10.1016/j.ufug.2015.07.009

[ece311461-bib-0031] Hill, M. , & Gauch, H. (1980). Detrended correspondence analysis: An improved ordination technique. Vegetatio, 42(1), 47–58.

[ece311461-bib-0032] Hölker, F. , Moss, T. , Griefahn, B. , Kloas, W. , Voigt, C. C. , Henckel, D. , Hänel, A. , Kappeler, P. M. , Völker, S. , Schwope, A. , Franke, S. , Uhrlandt, D. , Fischer, J. , Klenke, R. , Wolter, C. , & Tockner, K. (2010). The dark side of light: A transdisciplinary research agenda for light pollution policy. Ecology and Society, 15(4), 13. 10.5751/ES-03685-150413

[ece311461-bib-0033] Ibáñez‐Álamo, J. D. , Rubio, E. , Benedetti, Y. , & Morelli, F. (2017). Global loss of avian evolutionary uniqueness in urban areas. Global Change Biology, 23(8), 2990–2998. 10.1111/gcb.13567 27859999

[ece311461-bib-0034] Imai, H. , & Nakashizuka, T. (2010). Environmental factors affecting the composition and diversity of avian community in mid‐ to late breeding season in urban parks and green spaces. Landscape and Urban Planning, 96(3), 183–194. 10.1016/j.landurbplan.2010.03.006

[ece311461-bib-0035] Ives, C. D. , Lentini, P. E. , Threlfall, C. G. , Ikin, K. , Shanahan, D. F. , Garrard, G. E. , Bekessy, S. A. , Fuller, R. A. , Mumaw, L. , Rayner, L. , Rowe, R. , Valentine, L. E. , & Kendal, D. (2016). Cities are hotspots for threatened species. Global Ecology and Biogeography, 25(1), 117–126. 10.1111/geb.12404

[ece311461-bib-0036] Jaccard, P. (1902). Lois de distribution florale dans la zone alpine . https://www.e‐periodica.ch/digbib/view?pid=bsv‐002:1902:38::503

[ece311461-bib-0037] Jokimäki, J. (1999). Occurrence of breeding bird species in urban parks: Effects of park structure and broad‐scale variables. Urban Ecosystems, 3(1), 21–34.

[ece311461-bib-0039] La Sorte, F. A. , Lepczyk, C. A. , Aronson, M. F. J. , Goddard, M. A. , Hedblom, M. , Katti, M. , MacGregor‐Fors, I. , Mortberg, U. , Nilon, C. H. , Warren, P. S. , Williams, N. S. G. , & Yang, J. (2018). The phylogenetic and functional diversity of regional breeding bird assemblages is reduced and constricted through urbanization. Diversity and Distributions, 24(7), 928–938. 10.1111/ddi.12738

[ece311461-bib-0040] Magneville, C. , Loiseau, N. , Albouy, C. , Casajus, N. , Claverie, T. , Escalas, A. , Leprieur, F. , Maire, E. , Mouillot, D. , & Villéger, S. (2022). mFD: An R package to compute and illustrate the multiple facets of functional diversity. Ecography, 2022(1), 1–15.

[ece311461-bib-0042] Marzluff, J. M. (2017). A decadal review of urban ornithology and a prospectus for the future. Ibis, 159(1), 1–13. 10.1111/ibi.12430

[ece311461-bib-0043] Matuoka, M. A. , Benchimol, M. , Almeida‐Rocha, J. M. d. , & Morante‐Filho, J. C. (2020). Effects of anthropogenic disturbances on bird functional diversity: A global meta‐analysis. Ecological Indicators, 116, 1–9. 10.1016/j.ecolind.2020.106471

[ece311461-bib-0044] McKinney, M. L. (2002). Urbanization, biodiversity, and conservation. Bioscience, 52(10), 883–890. 10.1641/0006-3568(2002)052[0883:UBAC]2.0.CO;2

[ece311461-bib-0045] Moll, R. J. , Cepek, J. D. , Lorch, P. D. , Dennis, P. M. , Tans, E. , Robison, T. , Millspaugh, J. J. , & Montgomery, R. A. (2019). What does urbanization actually mean? A framework for urban metrics in wildlife research. Journal of Applied Ecology, 56(5), 1289–1300. 10.1111/1365-2664.13358

[ece311461-bib-0046] Morelli, F. , Mikula, P. , Benedetti, Y. , Bussière, R. , & Tryjanowski, P. (2018). Cemeteries support avian diversity likewise urban parks in European cities: Assessing taxonomic, evolutionary and functional diversity. Urban Forestry & Urban Greening, 36, 90–99. 10.1016/j.ufug.2018.10.011

[ece311461-bib-0047] Oke, T. R. (1973). City size and the urban heat island. Atmospheric Environment, 7, 769–779.

[ece311461-bib-0048] Oksanen, J. , Simpson, G. L. , Blanchet, F. G. , Kindt, R. , Legendre, P. , Minchin, P. R. , O'Hara, R. B. , Solymos, P. , Stevens, M. H. H. , Szoecs, E. , Wagner, H. , Barbour, M. , Bedward, M. , Bolker, B. , Borcard, D. , Carvalho, G. , Chirico, M. , Caceres, M. D. , Durand, S. , … Weedon, J. (2022). *vegan: Community ecology package* [Internet]. https://CRAN.R‐project.org/package=vegan

[ece311461-bib-0049] Oliveira Hagen, E. , Hagen, O. , Ibáñez‐Álamo, J. D. , Petchey, O. L. , & Evans, K. L. (2017). Impacts of urban areas and their characteristics on avian functional diversity. Frontiers in Ecology and Evolution, 5, 84. 10.3389/fevo.2017.00084

[ece311461-bib-0050] O'Neal, C. M. (2006). Urban parks as shared spaces? The utility of alert distances as indicators of avian tolerance of humans in Stirling, Scotland. Area, 38(3), 301–311. 10.1111/j.1475-4762.2006.00695.x

[ece311461-bib-0051] Palacio, F. X. (2020). Urban exploiters have broader dietary niches than urban avoiders. Ibis, 162(1), 42–49. 10.1111/ibi.12732

[ece311461-bib-0052] Palacio, F. X. , Ibañez, L. M. , Maragliano, R. E. , & Montalti, D. (2018). Urbanization as a driver of taxonomic, functional, and phylogenetic diversity losses in bird communities. Canadian Journal of Zoology, 96(10), 1114–1121. 10.1139/cjz-2018-0008

[ece311461-bib-0053] Patankar, S. , Jambhekar, R. , Suryawanshi, K. R. , & Nagendra, H. (2021). Which traits influence bird survival in the city? A review. Land, 10(2), 92. 10.3390/land10020092

[ece311461-bib-0054] Paul, M. J. , & Meyer, J. L. (2001). Streams in the urban landscape. Annual Review of Ecology and Systematics, 32(1), 333–365.

[ece311461-bib-0055] Pickett, S. T. A. , Cadenasso, M. L. , Grove, J. M. , Boone, C. G. , Groffman, P. M. , Irwin, E. , Kaushal, S. S. , Marshall, V. , McGrath, B. P. , Nilon, C. H. , Pouyat, R. V. , Szlavecz, K. , Troy, A. , & Warren, P. (2011). Urban ecological systems: Scientific foundations and a decade of progress. Journal of Environmental Management, 92(3), 331–362. 10.1016/j.jenvman.2010.08.022 20965643

[ece311461-bib-0056] Planillo, A. , Kramer‐Schadt, S. , Buchholz, S. , Gras, P. , von der Lippe, M. , & Radchuk, V. (2021). Arthropod abundance modulates bird community responses to urbanization. Diversity and Distributions, 27(1), 34–49. 10.1111/ddi.13169

[ece311461-bib-0057] R Core Team . (2023). R: A language and environment for statistical computing [internet]. R Foundation for Statistical Computing. https://www.R‐project.org/

[ece311461-bib-0058] Ram, K. , & Wickham, H. (2018). *wesanderson: A Wes Anderson palette generator* [Internet]. https://CRAN.R‐project.org/package=wesanderson

[ece311461-bib-0059] Ramalho, C. E. , & Hobbs, R. J. (2012). Time for a change: Dynamic urban ecology. Trends in Ecology & Evolution, 27(3), 179–188. 10.1016/j.tree.2011.10.008 22093191

[ece311461-bib-0060] Ren, K. , & Russell, K. (2021). *formattable: Create “formattable” data structures* [Internet]. https://CRAN.R‐project.org/package=formattable

[ece311461-bib-0061] Sandström, U. G. , Angelstam, P. , & Mikusiński, G. (2006). Ecological diversity of birds in relation to the structure of urban green space. Landscape and Urban Planning, 77(1–2), 39–53. 10.1016/j.landurbplan.2005.01.004

[ece311461-bib-0062] Scharon, J. (2010). Ergebnisse der Untersuchung der Brutvögel in ausgewählten Parkanlagen Berlins [Internet]. NABU Berlin. https://naturschutz‐und‐denkmalpflege.projekte.tu‐berlin.de/media/pdf/Brutvoegel_Scharon_2010.pdf

[ece311461-bib-0063] Scharon, J. (2015). Großer Tiergarten in Berlin: Wandel der Vogelwelt. Der Falke, 62, 4.

[ece311461-bib-0064] Scharon, J. (2020). Die Brutvögel des Großen Tiergarten 2010: Veränderungen gegenüber vorangegangenen Erfassungen. Berliner Ornithologische Berichte, 30, 25–48.

[ece311461-bib-0065] Schütz, C. , & Schulze, C. H. (2015). Functional diversity of urban bird communities: Effects of landscape composition, green space area and vegetation cover. Ecology and Evolution, 5(22), 5230–5239. 10.1002/ece3.1778 30151126 PMC6102532

[ece311461-bib-0066] Sekercioglu, C. (2006). Increasing awareness of avian ecological function. Trends in Ecology & Evolution, 21(8), 464–471. 10.1016/j.tree.2006.05.007 16762448

[ece311461-bib-0067] Sol, D. , Trisos, C. , Múrria, C. , Jeliazkov, A. , González‐Lagos, C. , Pigot, A. L. , Ricotta, C. , Swan, C. M. , Tobias, J. A. , & Pavoine, S. (2020). The worldwide impact of urbanisation on avian functional diversity. Ecology Letters, 23(6), 962–972. 10.1111/ele.13495 32266768

[ece311461-bib-0068] Sprötge, M. (1991). Die Vogelgemeinschaft des Großen Tiergartens in Berlin. Technische Universität Berlin, Landschaftsentwicklung und Umweltforschung (Schriftenreihe des Fachbereichs Landschaftsentwicklung der TU Berlin).

[ece311461-bib-0070] Thompson, R. , Tamayo, M. , & Sigurðsson, S. (2022). Urban bird diversity: Does abundance and richness vary unexpectedly with green space attributes? Journal of Urban Ecology, 8(1), 1–13. 10.1093/jue/juac017

[ece311461-bib-0071] Tobias, J. A. , Sheard, C. , Pigot, A. L. , Devenish, A. J. M. , Yang, J. , Sayol, F. , Neate‐Clegg, M. H. C. , Alioravainen, N. , Weeks, T. L. , Barber, R. A. , Walkden, P. A. , MacGregor, H. E. A. , Jones, S. E. I. , Vincent, C. , Phillips, A. G. , Marples, N. M. , Montaño‐Centellas, F. A. , Leandro‐Silva, V. , Claramunt, S. , … Schleuning, M. (2022). AVONET: Morphological, ecological, and geographical data for all birds. Ecology Letters, 25(3), 581–597. 10.1111/ele.13898 35199922

[ece311461-bib-0072] UBA (Ed.). (2019). Luftqualität 2018. Vorläufige Auswertung . 28.

[ece311461-bib-0073] UN DESA . (2019). The world's cities in 2018 – Data booklet [internet]. United Nations, Department of Economic and Social Affairs, Population Division. https://www.un‐ilibrary.org/population‐and‐demography/the‐world‐s‐cities‐in‐2016_8519891f‐en

[ece311461-bib-0074] Vitousek, P. M. , Mooney, H. A. , Lubchenco, J. , & Melillo, J. M. (1997). Human domination of Earth's. Ecosystems, 277, 7–499.

[ece311461-bib-0075] Wendland, F. (1993). Der Große Tiergarten in Berlin – Seine Geschichte und Entwicklung in fünf Jahrhunderten. Gebrüder Mann Verlag.

[ece311461-bib-0076] Wickham, H. (2016). *ggplot2: Elegant graphics for data analysis* [Internet]. https://ggplot2.tidyverse.org

[ece311461-bib-0077] Wickham, H. (2023). *stringr: Simple, consistent wrappers for common string operations* [Internet]. https://CRAN.R‐project.org/package=stringr

[ece311461-bib-0078] Wickham, H. , & Bryan, J. (2023). *readxl: Read excel files* [Internet]. https://CRAN.R‐project.org/package=readxl

[ece311461-bib-0079] Wickham, H. , François, R. , Henry, L. , Müller, K. , & Vaughan, D. (2023). *dplyr: A grammar of data manipulation* [Internet]. https://CRAN.R‐project.org/package=dplyr

[ece311461-bib-0080] Wickham, H. , Averick, M. , Bryan, J. , Chang, W. , D'Agostino McGowan, L. , François, R. , Grolemund, G. , Hayes, A. , Henry, L. , Hester, J. , Kuhn, M. , Pedersen, T. L. , Miller, E. , Stephan, M. B. , Müller, K. , Ooms, J. , Robinson, D. , Seidel, D. P. , Spinu, V. , … Yutani, H. (2019). Welcome to the tidyverse. Journal of Open Source Software, 4(43), 1686. 10.21105/joss.01686

[ece311461-bib-0081] Wilke, C. (2020). *cowplot: Streamlined plot theme and plot annotations for “ggplot2”* [Internet]. https://CRAN.R‐project.org/package=cowplot

[ece311461-bib-0082] Witt, K. , & Steiof, K. (2013). Rote Liste und Liste der Brutvögel von Berlin, 3. Fassung. Berliner Ornithologische Berichte, 23, 24.

[ece311461-bib-0083] Yang, M. , Callaghan, C. T. , & Wu, J. (2023). How do birds with different traits respond to urbanization? A phylogenetically controlled analysis based on citizen science data and a diverse urbanization measurement. Landscape and Urban Planning, 237, 1–12. 10.1016/j.landurbplan.2023.104801

